# Influence of exposure differences on city-to-city heterogeneity in PM_2.5_-mortality associations in US cities

**DOI:** 10.1186/s12940-016-0208-y

**Published:** 2017-01-04

**Authors:** Lisa K. Baxter, James L. Crooks, Jason D. Sacks

**Affiliations:** 1 National Health and Environmental Effects Research Laboratory, United States Environmental Protection Agency, 109 T.W. Alexander Drive, Research Triangle Park, NC 27711 USA; 2 National Center for Environmental Assessment, United States Environmental Protection Agency, 109 T.W. Alexander Drive, Research Triangle Park, NC 27711 USA; 3 Present address: Division of Biostatistics and Bioinformatics and Department of Biomedical Research, National Jewish Health, 1400 Jackson St., Denver, CO 80206 USA; 4 Department of Epidemiology, Colorado School of Public Health, 13001 E. 7th Place, Aurora, CO 80045 USA

**Keywords:** Particulate matter, Epidemiology, Exposure, Meta-regression, Cluster analysis

## Abstract

**Background:**

Multi-city population-based epidemiological studies have observed heterogeneity between city-specific fine particulate matter (PM_2.5_)-mortality effect estimates. These studies typically use ambient monitoring data as a surrogate for exposure leading to potential exposure misclassification. The level of exposure misclassification can differ by city affecting the observed health effect estimate.

**Methods:**

The objective of this analysis is to evaluate whether previously developed residential infiltration-based city clusters can explain city-to-city heterogeneity in PM_2.5_ mortality risk estimates. In a prior paper 94 cities were clustered based on residential infiltration factors (e.g. home age/size, prevalence of air conditioning (AC)), resulting in 5 clusters. For this analysis, the association between PM_2.5_ and all-cause mortality was first determined in 77 cities across the United States for 2001–2005. Next, a second stage analysis was conducted evaluating the influence of cluster assignment on heterogeneity in the risk estimates.

**Results:**

Associations between a 2-day (lag 0–1 days) moving average of PM_2.5_ concentrations and non-accidental mortality were determined for each city. Estimated effects ranged from −3.2 to 5.1% with a pooled estimate of 0.33% (95% CI: 0.13, 0.53) increase in mortality per 10 μg/m^3^ increase in PM_2.5_. The second stage analysis determined that cluster assignment was marginally significant in explaining the city-to-city heterogeneity. The health effects estimates in cities with older, smaller homes with less AC (Cluster 1) and cities with newer, smaller homes with a large prevalence of AC (Cluster 3) were significantly lower than the cluster consisting of cities with older, larger homes with a small percentage of AC.

**Conclusions:**

This is the first study that attempted to examine whether multiple exposure factors could explain the heterogeneity in PM_2.5_-mortality associations. The results of this study were found to explain a small portion (6%) of this heterogeneity.

**Electronic supplementary material:**

The online version of this article (doi:10.1186/s12940-016-0208-y) contains supplementary material, which is available to authorized users.

## Background

Multi-city population-based epidemiological studies of short-term PM_2.5_ exposures and mortality have provided evidence of heterogeneity in risk estimates between communities and cities [1, 2]. The inability to explain the city-to-city heterogeneity, both nationally and within a region, in PM_2.5_ mortality risk estimates observed in multi-city studies remains a key uncertainty in the examination of the relationship between short-term PM_2.5_ exposures and mortality. One potential reason for these differences is the use of ambient monitors, such as those reported in the United States Environmental Protection Agency’s Air Quality System, as a surrogate for exposure. These fixed site monitors are often sited for regulatory and attainment purposes, so they are not optimal for obtaining representative exposures for individuals or groups with health concern. This may introduce bias into the observed risk estimates if the relationship between ambient monitor measurements and personal exposure estimates varies by city [3].

Exposures often vary in space and time due to an individual’s activities. For example, an individual’s exposure will vary depending on the location of their home, work or school, as well as their commuting patterns [4]. People also spend the majority of their time indoors and where ambient PM_2.5_ can readily penetrate [5, 6]. This results in a substantial portion of an individual’s exposure to ambient PM_2.5_ occurring while indoors. Although individual activities vary within a day, ambient monitors are used to assign exposure in epidemiologic studies and this has been demonstrated to be an appropriate exposure surrogate due to ambient concentrations being well correlated with the ambient component of PM_2.5_ personal exposure [7]. However, when focusing on the composition of PM_2.5_ it is quite possible it may differ from the composition measured at the ambient monitor compared to other near-source environments, such as in-vehicles. The observed city-to-city heterogeneity has not been clearly linked to any one PM_2.5_ component or source [8], nor has there been evidence that the city specific relationship between ambient concentrations of PM_2.5_ components and gaseous pollutants with PM_2.5_ mass explains any city-to-city heterogeneity [9]. This has led to the hypothesis that exposure patterns (i.e., indoor and outdoor) may explain some of the heterogeneity in risk estimates observed in PM_2.5_-mortality studies.

Factors that differ between communities could be significant effect modifiers of the PM_2.5_-mortality association and may explain some of the heterogeneity across cities [10]. In a previous analysis cities were clustered with similar exposure distributions based on residential infiltration and in-vehicle commuting characteristics [3]. These characteristics included percent of homes with central air conditioning (AC), year home was built, home size, and in-vehicle commuting times and distances. The objective of this analysis is to determine whether these previously developed clusters can help explain city-to-city heterogeneity in PM_2.5_ mortality risk estimates. In this study, the association between PM_2.5_ and daily non-accidental mortality in 77 Core-Based Statistical Areas (CBSAs) is examined across the continental United States between 2001 and 2005. A CBSA is a geographic area that consists of one or more counties anchored by an urban center of at least 10,000 people plus adjacent counties that are socioeconomically tied to the urban center by commuting [11]. For the remainder of this paper the term “city” will be used in place of CBSA. Effect modification by cluster will be examined to determine whether these city-varying characteristics result in differential mortality associations with PM_2.5_.

## Methods

The association between daily PM_2.5_ concentrations and non-accidental mortality was determined for 77 cities across the continental United States for the years 2001–2005 using Poisson time-series models. The definitions for the 77 cities in this paper are based on the CBSAs of the White House Office of Management and Budget [12]. Health events were sorted into multi-county metropolitan regions centered on existing air quality monitors according to either county of event or county of residence. Once city-specific risk estimates were obtained, cities were grouped into clusters based on the approach outlined in [3]. Meta-regression was applied to examine the cluster assignment, and the individual characteristics (percent of homes with central AC, mean year home was built, and mean home size) used to develop the clusters.

### Mortality data

Individual-level mortality data for the entire U.S. from 2001 through 2005 were obtained through the National Center for Health Statistics from administrative systems for vital event records maintained by State and local health departments (http://www.cdc.gov/nchs/about.htm). All mortality data used in this study provide non-confidential information on individuals including state of death, county of death, age, gender, date of death and primary cause of death. For this study, we examined only those individuals who died of non-accidental causes (10th revision ICD codes (ICD10) U01-Y98 were excluded). Three age groups were defined: 0 to 64 years, 65 to 74 years and 75 years and older.

### Air pollution and meteorological data

The air pollution data was retrieved from the EPA’s Air Quality System Technology Transfer Network [13] which provides daily and hourly PM_2.5_ concentrations from the EPA’s National and State Local Ambient Monitoring Stations. There are typically multiple monitors located within a city with some monitors providing integrated daily measurements and others providing continuous hourly measurements of PM_2.5_. We focused on integrated daily measurements since the mortality data were only available at a daily time resolution.

When more than one PM_2.5_ monitor was available for a city it was necessary to determine which monitors represented the general population’s exposure over the desired area. First, all values from a given monitor which operated less than 6 months or had fewer than 30 observations were deleted. Next, correlation coefficients were computed for each pair of monitors within the county. All values in which a monitor was deemed uncorrelated with its neighboring monitors were deleted. These monitors most likely measured a local pollution source that would not represent the general population exposure over the entire city. A monitor was considered uncorrelated if it had a correlation of <0.8 with the majority of the monitors within that city.

Once appropriate monitors were identified, it was necessary to determine a summary measure of PM_2.5_ concentrations over the county. As the sampling schedule differed by monitor, with some having daily and others having measurements every 3–6 days, taking a simple average across multiple monitors would capture variation in monitor measurement patterns rather than the true variability in concentrations across the days measured in the county [2]. The PM_2.5_ values were averaged using the following equations. First, a global mean (*m*) and variance (*v*) was created within a city shown in Eqs. (1) and (2), respectively.

1 $$ m=\frac{1}{N}{\displaystyle {\sum}_{i=1}^n{{\displaystyle {\sum}_{t=1}^Tx}}_{i,t}} $$

2 $$ v=\frac{1}{N-1}{\displaystyle {\sum}_{i=1}^n{\displaystyle {\sum}_{t=1}^T{\left({x}_{i,t}-m\right)}^2}} $$

Where *N* is the total number of measured values within a given city for the entire time period, *n* is the number of monitors within a city, *T* is the total number of days a given monitor recorded measured values, and *x*_*t*,*i*_ is the daily PM_2.5_ value on day *t* at location *i*.

Next all values (*s*_*i,t*_) were standardized as illustrated in Eq. (3).

3 $$ {s}_{i,t}=\frac{x_{i,t}-m}{\sqrt{v}} $$

Eq. (4) calculates the average within a given day (*d*_*t*_)

4 $$ {d}_t=\frac{1}{n}{\displaystyle {\sum}_{i=1}^n{s}_{i,t}} $$

Finally the standardization of the daily value was reversed to calculate an average daily PM_2.5_ concentration for each city (*x*_*t*_) (Eq. (5)).

5 $$ {x}_t={d}_t\sqrt{v}+m $$

Meteorological data for all U.S. cities was obtained from the U.S. Department of Commerce’s National Climatic Data Center [14]. The daily data include 24-h averages of ambient temperature, dew point temperature, apparent temperature, relative humidity, barometric pressure; and total 24-h precipitation. If there was more than one monitor within a city these values were averaged across all monitors. Daily values of apparent temperature (T_ap_), a measure which reflects the temperature the body actually perceives, was derived from the dry bulb (T_db_) and dew point (T_dp_) temperatures using the following Eq. (6) (all temperatures in Celsius) [2]:

6 $$ {\mathrm{T}}_{\mathrm{ap}} = -2.653 + 0.994{\mathrm{T}}_{\mathrm{db}} + 0.0153{{\mathrm{T}}_{\mathrm{dp}}}^2 $$

### Cluster analysis

The methods used to identify clusters and the results of this analysis have been discussed in a previous paper [3]. Briefly, in Baxter et al. [3] clusters represented cities with similar exposure distributions based on residential infiltration and in-vehicle commuting characteristics. Factors related to residential infiltration and commuting were developed from the American Housing Survey from 2001 to 2005 for 94 cities. These cities all had populations over 100,000. Two separate cluster analyses using a k-means clustering algorithm were conducted to cluster cities based on these factors. The first analysis only included residential infiltration factors (i.e. percent of homes with central AC, mean year home was built, and mean home size) while the second incorporated both infiltration and commuting (i.e. mean in-vehicle commuting time and mean in-vehicle commuting distance) factors. The focus of this analysis is only on the clusters based on residential infiltration factors as the results from the combination of infiltration and commuting factors resulted in too many clusters with a small number of cities in each cluster.

Clustering on residential infiltration factors resulted in 5 clusters, with two having distinct exposure distributions. Table 1 presents the residential infiltration factors by cluster.

**Table 1:** Characteristics of residential infiltration factors by cluster (source Baxter et al. [3])

	Cluster 1(*N* ^a^ = 24)	Cluster 2 (*N* = 5)	Cluster 3 (*N* = 40)	Cluster 4 (*N* = 18)	Cluster 5 (*N* = 7)
	Mean (SD)	Mean (SD)	Mean (SD)	Mean (SD)	Mean (SD)
% of homes with central air conditioning	27.4 (13.7)	72.1 (18.8)	71.5 (9.6)	55.7 (10.4)	18.9 (5.6)
Mean year home was built	1954 (9.1)	1989 (8.8)	1970 (4.8)	1959 (4.7)	1945 (4.7)
Mean size of home (sq ft)	1672 (170.2)	2208 (224.4)	1662 (121.3)	2098 (188)	2253 (292)

^a^Number of cities

Cluster 1 consisted of cities with older, smaller homes with less central AC while homes in Cluster 2 cities were newer, larger, and more likely to have central AC. For the remaining clusters, cluster 3 represents cities with high prevalence of central AC with newer and smaller homes; cluster 4 represents cities with moderate prevalence of central AC with older and larger homes; and cluster 5 represents cities with low prevalence of central AC with older and larger homes. Additional file 1: Table S1 lists the cities s by cluster.

### Statistical analysis

Of the 94 cities examined in Baxter et al. [3], only those cities with more than 500 observation days for PM_2.5_ were included in the current analysis, resulting in a total of 77 cities. Single city PM_2.5_-mortailty risk estimates for each of the 77 cities were determined using Poisson regression analysis. Mortality counts were aggregated by date of event and age group within each city, and matched with PM_2.5_ and meteorological data by date and city. These data were then analyzed to estimate the association between daily PM_2.5_ concentrations and daily mortality events while adjusting for time-varying confounders. Specifically, the single city time-series models were fit using the glm function in R assuming quasi-Poisson-distributed responses and the log link function. The linear predictor included a separate intercept for each age group, a linear effect of 2-day moving average (lag 0–1 days) PM_2.5_ concentration chosen based on previous studies [15], a nonlinear effect of time represented by a natural spline with 6 degrees of freedom per year to account for seasonal and longer-term trends in mortality, and nonlinear effects of same day temperature, 1-day lagged temperature, and dew-point temperature, represented in each case by a natural spline with 3 degrees of freedom.

The second stage of this analysis involved obtaining a pooled estimate using a meta-analysis of the city-specific estimates and their standard errors. The degree of heterogeneity in the pooled effect estimate over the cities was determined using a random effects meta-analysis to obtain the heterogeneity variance component. This component represents the true heterogeneity in response, above what would be expected by the stochastic variability in the estimates [2]. To test for significant heterogeneity between city-specific risk estimates, which would indicate whether random effects were necessary in the meta-regression, a Q-statistic was computed. It was hypothesized that the aforementioned clusters could be significant effect modifiers of the PM_2.5_-mortality associations and may explain some of the city-to-city heterogeneity. Thus, meta-regression was applied using the clusters. All data was managed using R [16] and SAS 9.4 [17]. In addition to using the clusters as the independent variable, the individual city characteristics (i.e. home age, home size, and presence of AC) used to develop the clusters were also examined.

## Results

**Fig. 1:**
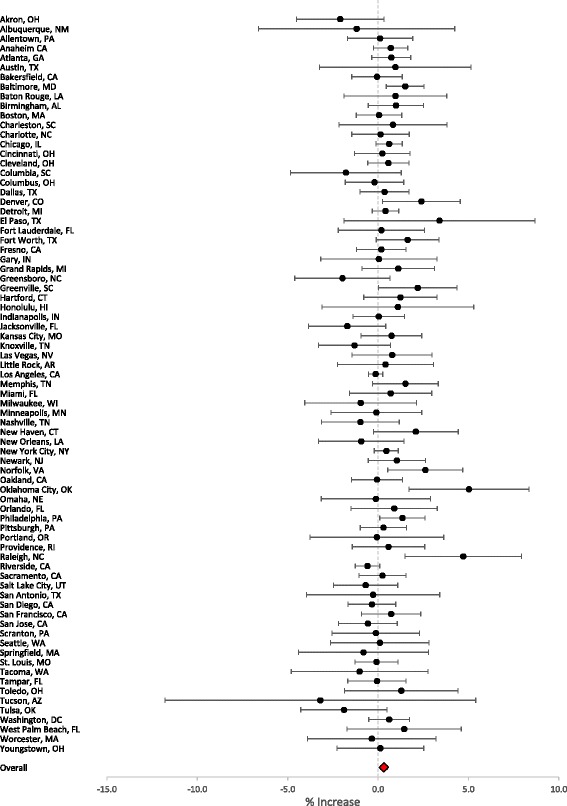
Percent increase in non-accidental mortality with a 10 μg/m3 increase in PM2.5 concentrations using a moving average of the previous 2-days (lag 0–1) (circles = estimate; whiskers = 95% confidence interval)

Associations between a 2-day (lag 0–1 days) moving average of PM_2.5_ concentrations and non-accidental mortality were determined for each city. Figure 1 presents the percent increase in non-accidental mortality with a 10 μg/m^3^ increase in 24-h average PM_2.5_ concentrations. Results ranged from −3.2 to 5.1%. After pooling the city-specific effect estimates into an overall effect estimate, short-term PM_2.5_ exposure was found to increase daily non-accidental mortality by 0.33% (95% CI: 0.13, 0.53). A Q-statistic was computed and compared to *χ* ^2^ distribution with 76° of freedom. Significant heterogeneity was observed among city-specific effect estimates (Q-statistic *P* = 0.1).

The PM_2.5_ summary data for each cluster is presented in Table 2. Mean 24-h average PM_2.5_ concentrations were marginally significantly different between clusters (Kruskal Wallis test, *p* = 0.09). The list of cities within each cluster assignment from Baxter et al. [3] is presented in Additional file 1: Table S1.

**Table 2:** Summary statistics of ambient PM_2.5_ concentrations (μg/m^3^) by cluster

Cluster	N^a^	Mean (SD)	Range
1	16	13.0 (3.32)	4.14–19.9
2	5	13.6 (3.14)	8.66–16.2
3	34	12.2 (3.5)	6.33–22.7
4	17	14.1 (2.02)	10.2–16.6
5	5	13.7 (1.2)	12.2–14.9

^a^Number of cities

Summary statistics for the percent increase in non-accidental mortality with a 10 μg/m^3^ increase in PM_2.5_ concentration by cluster are shown in Table 3. Clusters 4 and 5 had the highest percent increase in non-accidental mortality while Cluster 1 had the lowest. Of note clusters 2 and 5 consist of only 5 cities reducing the confidence in these results. Although the mean percent increase was positive across all clusters, except cluster 1, the range in the percent increase across cities within clusters tended to encompass both negative and positive values.

**Table 3:** Percent increase in non-accidental mortality for a 10 μg/m^3^ increase in 24-h average PM_2.5_ concentrations, lag 0-1

Cluster	N^a^	Pooled estimate	95% CI
1	16	−0.01	−0.31–0.29
2	5	0.41	−0.28–1.1
3	34	0.25	−0.15–0.65
4	17	0.66	0.35–0.97
5	5	0.50	−0.27–1.3

^a^Number of cities

A second stage analysis was conducted in which the betas for each individual city were weighted by their corresponding standard errors and regressed against each categorical cluster variable resulting in a *p*-value of 0.07 and an R^2^ of 6%. This indicates that the cluster assignment was marginally significant in terms of explaining the city-to-city heterogeneity in PM_2.5_-mortality associations. Results from each cluster were contrasted against one another to determine which clusters were significantly different from one another. The health effects estimates in Cluster 1 and Cluster 3 were significantly lower than those in Cluster 4. In addition to examining cluster assignment, second stage analyses on the individual factors (home age, home size, and presence of AC) were also conducted. Home size was the only significant modifier with larger homes resulting in larger risk coefficients.

## Discussion

There remains uncertainty to the cause of the observed heterogeneity in city-to-city PM_2.5_-mortality associations in U.S. based multi-city studies. Often this heterogeneity has been attributed to the regional and national variation in components of PM_2.5_; however, a clear difference in the air pollution mixtures has yet to be identified. A previous study examining city-to-city differences in ambient concentrations and the relationship between PM_2.5_ components and gaseous pollutants with PM_2.5_ mass did not provide any evidence of clear differences in ambient concentrations or sources between cities [9]. Additionally, the evidence from epidemiologic and toxicological studies has not demonstrated that any one component or source is more strongly related with specific health outcomes [8, 18, 19].

Infiltration, defined as the fraction of the outdoor concentration that penetrates indoors and remains suspended, varies between cities, between homes, and over time within homes [20]. Allen at al. developed models to predict infiltration based on behavioral factors such as air conditioning use and windows opening that can vary seasonally [21]. The wide variation observed in residential infiltration rates [21] supports the hypothesis that city-to-city differences in the personal exposure-ambient monitor relationship may also be contributing to the observed heterogeneity in PM_2.5_ mortality risk estimate across cities This has led to epidemiologic studies examining factors that may influence individual exposures and ultimately associations in studies of short-term air pollution exposures and various health outcomes (e.g., mortality), such as air exchange rate [22], infiltration rates [23], and air conditioning use [2]. Each of these studies has provided initial information on the importance of accounting for factors that may influence individual exposure, but the overall air pollution exposures people encounter are dictated by a variety of factors, not just one at a time. To further expand upon some of these initial studies, Baxter et al. [3] used information on individual exposure factors representative of infiltration and commuting to create clusters of cities with similar exposure profiles to examine whether different exposure profiles help explain the city-to-city heterogeneity in PM_2.5_-mortality risk estimates.

Building off the work detailed in Baxter et al. [3], the objective of this analysis was to determine whether the previously developed clusters help explain the city-to-city heterogeneity in PM_2.5_-mortality associations. Overall, we reported a 0.33% increase in non-accidental mortality for a 10 μg/m^3^ increase in previous 2-day moving average (lag 0–1) PM_2.5_ concentrations for 77 U.S. cities for the years of 2001–2005. The examination of cluster assignment was found to be marginally significant in explaining the heterogeneity in PM_2.5_-mortality associations. When comparing results between clusters, we only observed evidence of significant differences in mortality associations between Cluster 1 and 4 and Cluster 3 and 4, while the PM_2.5_-mortality associations in Cluster 1 and 3 are significantly smaller in magnitude compared to those in Cluster 4. As a sensitivity analysis, we performed a meta-regression on the individual factors that comprise the clusters and found that only home size appeared to explain the heterogeneity in the PM_2.5_-mortality associations, with larger associations in larger homes. However, in Baxter et al. [3] home size was not well correlated with the other exposure factors included in the infiltration exposure factors evaluations.

Upon closer examination, there are clear differences in the housing characteristics between clusters that appeared to contribute in explaining the heterogeneity in PM_2.5_ mortality associations. Air exchange rates have been found to be higher in larger and older homes [24] and in homes with less central AC due to more opening of windows [25] resulting in higher exposures to outdoor PM and associations larger in magnitude. Homes in Cluster 1 were on average 426 ft^2^ smaller, had 27% less central AC, and similar age homes compared to Cluster 4. Larger health effect were observed in Cluster 4 suggesting higher exposures in those homes. This is in agreement with the significant findings on home size in the sensitivity analyses with larger homes size associated with larger health effect estimates. Similarly, the underlying exposure profiles of Cluster 3 and 4 may help explain the difference in associations between the two clusters. Cluster 3 has a larger percentage of homes with central AC, as well as homes that are newer and smaller than those in Cluster 4. This difference between Clusters 3 and 4 would indicate that air exchange rates may be smaller in Cluster 3 resulting in lower exposures, which would subsequently result in associations smaller in magnitude for Cluster 3.

It is important to recognize that this study is subject to inherent limitations. One of the main limitations in the epidemiological analysis was the potential for exposure error from using an adjusted average of PM_2.5_ concentrations from a few monitors to characterize a population exposure in each of the cities. However, PM_2.5_ is relatively spatially homogenous and studies of personal exposures have shown that temporal variability in outdoor PM_2.5_ concentrations are a good surrogate for temporal variability in personal PM_2.5_ exposures [26, 27]. There is also potential for exposure error as the exposure factors that were used to generate the clusters are surrogates rather than direct measurements of residential infiltration. Furthermore, while reductions in residential infiltration will reduce exposures to PM_2.5_ of ambient origin it will also increase exposures to PM_2.5_ generated from indoor sources. This indoor PM_2.5_ may be independently associated with adverse health effects. Finally, a more thorough evaluation of potential differences between the five clusters examined in this study was limited by the small number of cities that comprised Clusters 2 and 5.

## Conclusions

Overall, this is the first study that attempted to examine whether multiple exposure factors could explain the heterogeneity in PM_2.5_-mortality associations. Not surprisingly, the results of this study only explain some of this heterogeneity as this is most likely due to a variety factors. In addition to the aforementioned differences in composition of the PM, variation in the city-specific PM_2.5_ mortality risk estimates could be due to differences in individual- or population-level characteristics between cities such as the age distribution of the population, the distribution of the population encompassing a certain socioeconomic status [2, 28]. In conclusion, this study demonstrates that multiple exposure factors should be considered in future endeavors to elucidate the underlying cause of the observed heterogeneity in PM_2.5_ mortality associations.

## Additional file

Additional file 1: Table S1. The lists of the CBSA by cluster that was produced from the cluster analysis conducted in the previous paper. (DOCX 16 kb)

## Abbreviations

AC: Air conditioning
CBSA: Core-based statistical area
PM_2.5_: Fine particulate matter
