# Supplementary material for: Influence of exposure differences on city-to-city heterogeneity in PM2.5-mortality associations in US cities
Source: Environ Health. 2017 Jan 4;16:1. doi: 10.1186/s12940-016-0208-y (PMC5209854; doi:10.1186/s12940-016-0208-y)
Supplement: Additional file 1: Table S1. — The lists of the CBSA by cluster that was produced from the cluster analysis conducted in the previous paper. (DOCX 16 kb) [file 12940_2016_208_MOESM1_ESM.docx]

Table S1. List of Cities by cluster for clustering analysis on residential infiltration factors

| Cluster 1 | Cluster 2 | Cluster 3 | Cluster 4 | Cluster 5 |
| --- | --- | --- | --- | --- |
| Madison, WI | Charlotte, NC | Charleston, SC | Greenville-Spartanburg, SC | Scranton-Wilkes Barre, PA |
| Colorado Springs, CO | Columbus, OH | Stockton, CA | Grand Rapids, MI | Syracuse, NY |
| Youngstown-Warrant, OH | Kansas City, MO-KS | McAllen-Edinburgh-Mission, TX | Lake County, IL | Allentown-Bethlehem-Easton, PA |
| Toledo, OH | Portland, OR | Wichita, KS | Salt Lake City-Ogden, UT | New Haven-Meriden, CT |
| Gary-Hammond, IN | Atlanta, GA | Sarasota, FL | Birmingham, AL | Boston, MA |
| Springfield, MA |  | Little Rock-North Little Rock, AK | Hartford, CT | Newark, NJ |
| Akron, OH |  | Knoxville, TN | Milwaukee, WI | Nassau-Suffolk, NY |
| Tacoma, WA |  | Greensboro-Winston Salem-High Point, NC | Cincinnati, OH-KY-IN |  |
| Worcester, MA |  | Columbia, SC | Cleveland, OH |  |
| Oxnard-Venture, CA |  | Bakersfield, CA | Denver, CO |  |
| Albany-Schenectady-Troy, NY |  | El Paso, TX | Pittsburgh, PA |  |
| Honolulu, HI |  | Baton Rouge, LA | Baltimore, MD |  |
| Bridgeport-Milford, CT |  | Albuquerque, NM | Saint Louis, MO-IL |  |
| Stamford, CT |  | Omaha, NE-IA | Minneapolis-Saint Paul, MN |  |
| Rochester, NY |  | Raleigh-Durham, NC | Philadelphia, PA-NJ |  |
| Buffalo, NY |  | Fresno, CA | Detroit, MI |  |
| Providence, RI |  | Tucson, AZ | Washington, DC-MD-VA |  |
| San Francisco, CA |  | Tulsa, OK | Chicago, IL |  |
| San Jose, CA |  | Oklahoma City, OK |  |  |
| Seattle, WA |  | Jacksonville, FL |  |  |
| Oakland, CA |  | West Palm Beach-Boca Raton, FL |  |  |
| San Diego, CA |  | Memphis, TN |  |  |
| New York City, NY |  | Austin, TX |  |  |
| Los Angeles-Long Beach, CA |  | Nashville, TN |  |  |
|  |  | New Orleans, LA |  |  |
|  |  | Las Vegas, NV |  |  |
|  |  | Indianapolis, IN |  |  |
|  |  | Norfolk-Virginia Beach, VA |  |  |
|  |  | Fort Lauderdale-Hollywood, FL |  |  |
|  |  | Orlando, FL |  |  |
|  |  | Fort Worth-Arlington, VA |  |  |
|  |  | San Antonio, TX |  |  |
|  |  | Sacramento,CA |  |  |
|  |  | Miami-Hialeah, FL |  |  |
|  |  | Tampa-Saint Petersburg-Clearwater, FL |  |  |
|  |  | Anaheim-Santa Ana, CA |  |  |
|  |  | Phoenix, AZ |  |  |
|  |  | San Bernardino-Riverside, CA |  |  |
|  |  | Dallas, TX |  |  |
|  |  | Houston, TX |  |  |
